# Is hypoimmunogenic stem cell therapy safe in times of pandemics?

**DOI:** 10.1016/j.stemcr.2022.02.014

**Published:** 2022-03-24

**Authors:** Friederike Matheus, Tal Raveh, Anthony E. Oro, Marius Wernig, Micha Drukker

**Affiliations:** 1 Institute for Stem Cell Research, iPSC Core Facility, Helmholtz Zentrum München GmbH, 85764 Neuherberg, Germany; 2 Institute for Stem Cell Biology and Regenerative Medicine, and Ludwig Center for Cancer Stem Cell Research, Stanford University School of Medicine, Stanford, CA, USA; 3 Program in Epithelial Biology, Stanford University School of Medicine, Stanford, CA 94305, USA; 4 Institute for Stem Cell Biology and Regenerative Medicine and Department of Pathology, Stanford University Medical School, Stanford, CA 94305, USA; 5 Division of Drug Discovery and Safety, Leiden Academic Centre for Drug Research (LACDR), Leiden University, 2333 CC RA Leiden, The Netherlands

## Abstract

The manipulation of human leukocyte antigens (HLAs) and immune modulatory factors in “universal” human pluripotent stem cells (PSCs) holds promise for immunological tolerance without HLA matching. This paradigm raises concerns should "universal" grafts become virally infected. Furthermore, immunological manipulation might functionally impair certain progeny, such as hematopoietic stem cells. We discuss the risks and benefits of hypoimmunogenic PSCs, and the need to further advance HLA matching and autologous strategies.

## Teaser

In this perspective, Matheus and colleagues discuss potential risks and limitations of “universal” grafts from hypoimmunogenic pluripotent stem cells. They raise concerns regarding the possibility of infection, e.g., by SARS-CoV-2, due to immunologically cloaked transplants, and consider the applicability of hypoimmunogenic cells in therapies necessitating functional MHC presentation, including hematopoietic stem cell, immunological, and cutaneous disorders.

## Main text

Progress in stem cell biology, bioengineering, and cell manufacturing, led by academic research and powered by a growing biotech sector, rapidly closes gaps for human pluripotent stem cell (PSC) therapies. Over 25 new human trials that utilize differentiated PSCs are on track for the clinical phase (Kobold et al., 2020). Since the manufacturing of autologous induced PSC grafts is currently very time consuming and costly, clinical-grade allogeneic PSCs, which are already available, can be used to lower matching barriers by modulation of major histocompatibility complex (MHC) expression (Deuse et al., 2019; Gornalusse et al., 2017; Han et al., 2019; Harding et al., 2019; Rong et al., 2014; Xu et al., 2019). The general premise of the hypoimmunogenic “universal” approach is to prevent the triggering of alloreactive host T cells by disabling the expression of their human leukocyte antigen (HLA) ligands, which are highly polymorphic. At the same time, immune checkpoint proteins such as programmed death-ligand 1 (PD-L1) or cluster of differentiation (CD) 47 can be ectopically expressed in order to further protect the transplanted cells from the recipient's T cells, natural killer (NK) cells, and macrophages (Deuse et al., 2019; Gornalusse et al., 2017). NK cells are particularly important in this regard because they get activated against cells lacking HLA-C proteins (Ichise et al., 2017). Despite the appealing concept of universal grafts that promise HLA matching-free transplantation for any individual, concerns have been voiced about the safety of such immunologically “cloaked” PSC transplants due to the potential formation of hypoimmunogenic cancers (Gonzalez et al., 2020). The first clinical trial based on universal cells was recently approved in Canada, and we therefore find it important to raise further safety considerations. In particular, caution must be exercised when manipulating the immunological surveillance of PSC transplants. Thus, we discuss the importance of intact MHC in the context of efficacious immune response against cells infected with viruses, and PSC therapies necessitating functional MHC for promoting immune protection.

Chronic and acute life-threatening infections in patients with genetic deficiencies of MHC class I and II, also known as bare lymphocyte syndrome (BLS) type I and II (Hanna and Etzioni, 2014), are indicators of potential risks associated with use of hypoimmunogenic PSC grafts. Because the key step in the generation of immune-evading PSCs is the knockout of *HLA* genes and/or *B2M* (Deuse et al., 2019; Gornalusse et al., 2017; Han et al., 2019), hypoimmunogenic tissues lacking HLA will not have the ability to induce cytotoxic CD8^+^ and helper CD4^+^ T cells by presentation of viral and other pathogen antigens should infection occur. Explicitly said, hypoimmunogenic cells could open the door to dramatic infection of the transplanted tissue and possibly surrounding tissues. A related idea is to downregulate MHC by knocking out genes in the antigen processing pathway, namely *TAP1* and/or *TAP2* (Cui et al., 2016). However, this could lead to hyperinflammation, such as in Wegener granulomatosis-like syndrome, where NK cells are aberrantly overactivated due to low HLA-I expression caused by *TAP1/TAP2* mutations (Moins-Teisserenc et al., 1999). Modified strategies that maintain partial antigen presentation, via HLA-C retention, for example, might be generally safer (Xu et al., 2019). Combining MHC depletion with ectopic expression of immunoregulatory suppressive genes such as B7-H1/PD-L1, B7-H2, CTLA-4-Ig, HLA-E, and CD47 will enhance hypoimmunity (Deuse et al., 2019; Gornalusse et al., 2017; Han et al., 2019; Harding et al., 2019; Rong et al., 2014) but will broadly suppress alloreactive immunity, NK cells, and macrophages (Mogensen, 2009), thus significantly increasing the possible risk of uncontrolled infection of the graft cells. A potentially safer approach involves transient induction of endogenous immunoinhibitory molecules. Recently, induction of PD-L1 was shown potential to protect PSC islets from T cells in immune-competent mice (Yoshihara et al., 2020), but it remains to be determined if this approach is effective for the long term and in humans, who contain significantly higher number of potentially alloreactive T cells.

More relevant to the coronavirus pandemic, severe acute respiratory syndrome coronavirus-2 (SARS-CoV-2) variants become increasingly contagious, evolve faster than updates of vaccines, and reinfection becomes the norm (Cohen and Burbelo, 2021; Singh et al., 2021). The likelihood that hypoimmunogenic cells will become infected with SARS-CoV-2, should they be used in the clinic, is therefore not negligible. In a worst-case scenario, SARS-CoV-2 infection might lead to destruction of immune-evading grafts and promote viral spreading to other organs. SARS-CoV-2 has a particularly broad tissue tropism combined with airborne transmission, which could pose a significant risk for the application of hypoimmunogenic grafts. This necessitates research toward the risks and benefits of hypoimmunogenic grafts in general and with respect to coronavirus disease 2019 (COVID-19) infection. In this context, inducible systems for controlled elimination of potentially infected or cancerous hypoimmunogenic PSCs after transplantation could be investigated (Harding et al., 2020; Martin et al., 2020).

A general limitation of hypoimmunogenic PSCs relates to tissues that require the MHC for building and maintaining immunity. This includes literally all lymphoid and myeloid lineages that differentiate from hematopoietic stem cells (HSCs). HSC transplantation is the most widely implemented stem cell therapy, with a decades-long track record in a multitude of clinical applications. Apart from the treatment of blood and bone marrow malignancies, the transplantation of HSCs serves to treat genetic forms of anemia, immunodeficiencies, immune dysregulation, neutropenia, and several metabolic disorders. Professional antigen-presenting cells (APCs), namely B cells, macrophages, and dendritic cells, as well as atypical MHC class II-expressing APCs such as type 3 innate lymphoid cells (ILC3s) (Kambayashi and Laufer, 2014), are progeny of HSCs. Therefore, hypoimmunogenic PSCs do not represent a suitable source for HSC therapies because of the requirement for intact MHC in adaptive and innate immunity. The same rationale applies to APCs derived directly from PSCs; for example, in prospective therapies for pulmonary alveolar proteinosis using PSC-derived macrophages (Shima et al., 2017). Keratinocytes are another example of cells whose function involves HLA-dependent antigen presentation and immunoregulation at the skin barrier, hence likely ruling out hypoimmunogenic PSCs as a source (Meister et al., 2015; Sebastiano et al., 2014). Thus, it is important to recognize that hypoimmunogenic PSCs might exclude applications of immune system cells.

Beyond this, there exists a possibility to induce tolerance toward virtually any differentiated tissue from PSCs based on the transplantation of HSCs from PSCs of the same donor. This involves mixed chimerism of the endogenous lymphohematopoietic system and the immune cells from PSCs-HSCs (Sachs et al., 2014). Tolerance induction is based on MHC-mediated depletion of graft-targeting recipient T cells by donor APCs (Ebens et al., 2020), which makes the process inconsistent when using hypoimmunogenic PSCs. In principle, it could be advantageous to replace endogenous hematopoiesis by PSC-HSC reconstitution for treating autoimmune disorders (Beilhack et al., 2003). Antibody-mediated conditioning for HSC engraftment to avoid chemotherapy and radiation risks is an important development for HSC engraftment including PSC-HSC (George et al., 2019). In this regard, the grafting of PSC-thymic epithelium could potentially support recipient T cell selection (Kwun et al., 2020). Since thymic T cell education is contingent on antigen presentation by MHC, use of hypoimmunogenic PSCs precludes therapies using thymic tissues. Similarly, PSC-thymic epithelium could be used to combat immunodeficiency caused by congenital thymic defects (Davies et al., 2017; Markert et al., 1999) or age-related thymic involution (Palmer et al., 2018), which could not be achieved using hypoimmunogenic PSCs. Overall, important potential therapies require immunologically intact PSC-derived cells for treating inborn and infection-associated immunological complications.

We conclude that immunological risks associated with the transplantation of hypoimmunogenic PSCs for solid organ grafts are potentially concerning, particularly during the ongoing COVID-19 pandemic. In terms of safety, infection risk is lower with PSC grafts with intact immunity. Furthermore, MHC proteins play critical roles in the immunological functions of HSC progeny, APCs, thymic epithelium, skin, and other types of cells, thus limiting prospects for the application of hypoimmunogenic universal PSCs in immunological, cutaneous, and likely additional disorders. We therefore propose two paths forward: first, efforts should be focused on technical innovation, such as robust automation of the reprogramming and differentiation procedures to commoditize autologous induced PSC (iPSC) manufacturing (Gagliano et al., 2019; Paull et al., 2015). Second, while such efforts are ongoing, HLA-homozygous iPSC lines could serve as “off-the-shelf” sources and are soon to enter clinical use in Japan (Morishima et al., 2020; Umekage et al., 2019). Also, transplantation of PSC-HSCs has potential to induce immune tolerance to grafts of other therapeutic cells derived from the same donor PSC. These approaches should allow PSCs to fulfill their vast potential as a remedy for a multitude of disorders.
